# Anesthetic-mediated cardioprotection: from molecular mechanisms to clinical translation challenges

**DOI:** 10.3389/fphys.2025.1688142

**Published:** 2025-09-17

**Authors:** Tingting Fu, Xiao Jia, Can Tang, Dan Yu, Hui Zhou, Xinghe Wang, Su Liu, Kunwei Wu

**Affiliations:** ^1^ Department of Rehabilitation Medicine and Geriatrics, Chongqing Liangping District People’s Hospital, Chongqing, China; ^2^ Jiangsu Province Key Laboratory of Anesthesiology, Jiangsu Province Key Laboratory of Anesthesia and Analgesia Application Technology, NMPA Key Laboratory for Research and Evaluation of Narcotic and Psychotropic Drugs, School of Anesthesiology, Xuzhou Medical University, Xuzhou, Jiangsu, China; ^3^ Department of Anesthesiology, The Affiliated Hospital of Xuzhou Medical University, Xuzhou, Jiangsu, China

**Keywords:** general anesthesia, propofol, ketamine, isoflurane, cardiology

## Abstract

Anesthetics have long been recognized as essential pharmacological agents for surgical procedures, primarily valued for their ability to induce unconsciousness and provide analgesia. However, emerging research over the past 3 decades has revealed an additional and potentially transformative property of certain anesthetics: their ability to protect the heart against ischemic injury. This comprehensive review examines the cardioprotective effects of both intravenous and volatile anesthetics, with particular focus on propofol, ketamine, isoflurane, and sevoflurane. We analyze the molecular mechanisms underlying their protective actions, including modulation of mitochondrial function, reduction of oxidative stress, and regulation of key survival pathways such as PI3K/Akt/GSK3βand p53 signaling. The review evaluates preclinical evidence from cellular and animal models, as well as clinical studies investigating anesthetic-mediated cardioprotection in cardiac surgery patients. Special attention is given to the phenomenon of anesthetic preconditioning and postconditioning, their comparative efficacy, and the challenges in translating these protective strategies into clinical practice. We also discuss emerging concepts such as the role of microRNAs in mediating anesthetic-induced protection and the potential cardioprotective benefits of anesthetic combinations. Finally, we identify critical gaps in current knowledge and propose future research directions that may enhance the clinical application of anesthetic-mediated cardioprotection.

## 1 Introduction

The recognition that anesthetic agents may confer cardioprotective benefits has fundamentally transformed our perspective on these pharmacological compounds (Lotz and Kehl, 2015). While anesthetics have been employed clinically since the mid-19th century, their capacity to actively safeguard the heart against ischemic injury has only emerged as a focus of rigorous scientific investigation in recent decades. This paradigm shift has created new opportunities to enhance outcomes in cardiac surgery and acute coronary syndrome management (Hendrix and Kramer, 2025).

The understanding of anesthetic-mediated cardioprotection stems from groundbreaking research on ischemic preconditioning, where scientists first demonstrated the heart’s remarkable ability to develop resistance to prolonged ischemia following brief ischemic episodes (Murry et al., 1986). This fundamental discovery unveiled the myocardium’s innate protective mechanisms and their potential for pharmacological stimulation. Further investigations revealed that specific anesthetic compounds could mimic these protective effects without requiring actual ischemic events, transforming our approach to both cardiovascular research and clinical anesthesia (Zhu et al., 2017; Shirakawa et al., 2014; Kobayashi et al., 2008; Ko et al., 1997a). Despite significant advances in medical technology, ischemia-reperfusion injury continues to pose major clinical challenges, particularly in cardiac surgery and acute coronary care settings (Xiang et al., 2024). The persistent occurrence of myocardial damage during procedures involving cardiopulmonary bypass remains a critical factor affecting postoperative recovery (Algoet et al., 2023). This ongoing clinical challenge highlights the importance of exploring anesthetic agents as potential therapeutic tools to reduce ischemia-reperfusion injury, offering a practical approach to improving patient outcomes using well-established medications (Kato and Foex, 2002).

This review offers a systematic examination of anesthetic-induced cardioprotection with three principal aims: first, to delineate the molecular mechanisms underlying the protective effects of various anesthetic agents; second, to assess the comparative efficacy of different anesthetics in both experimental and clinical settings; and third, to analyze the translational challenges and opportunities for optimizing patient outcomes. Our analysis focuses on propofol, ketamine, and volatile anesthetics (isoflurane, sevoflurane), which represent the most extensively studied agents in cardioprotection research. Building upon previous comprehensive evaluations (De Hert et al., 2005; Pagel and Crystal, 2018), this review provides an updated, comprehensive synthesis that significantly advances previous analyses. We integrate contemporary preclinical findings from cellular and animal models with robust clinical evidence, while addressing critical translational gaps identified in earlier reviews (Lin et al., 2021; Van Allen et al., 2012; Chiari and Fellahi, 2024; Tamura et al., 2025). Specifically, we examine patient-specific considerations (e.g., diabetes, aging) and propose practical strategies for clinical implementation. By emphasizing emerging biomarkers, combination therapies, and individualized application, this synthesis not only updates but significantly expands the current understanding of anesthetic-mediated cardioprotection.

The potential clinical impact of anesthetic cardioprotection is substantial. Successful translation of these effects could improve outcomes in cardiac surgery, enhance management of acute coronary syndromes, and potentially inform novel approaches to heart failure prevention. Furthermore, elucidation of these protective mechanisms may guide development of new pharmacological agents that provide cardioprotection independent of anesthetic effects, potentially benefiting non-surgical patients at risk of ischemic heart disease.

## 2 Mechanisms of anesthetic-induced cardioprotection

### 2.1 Propofol

**FIGURE 1:**
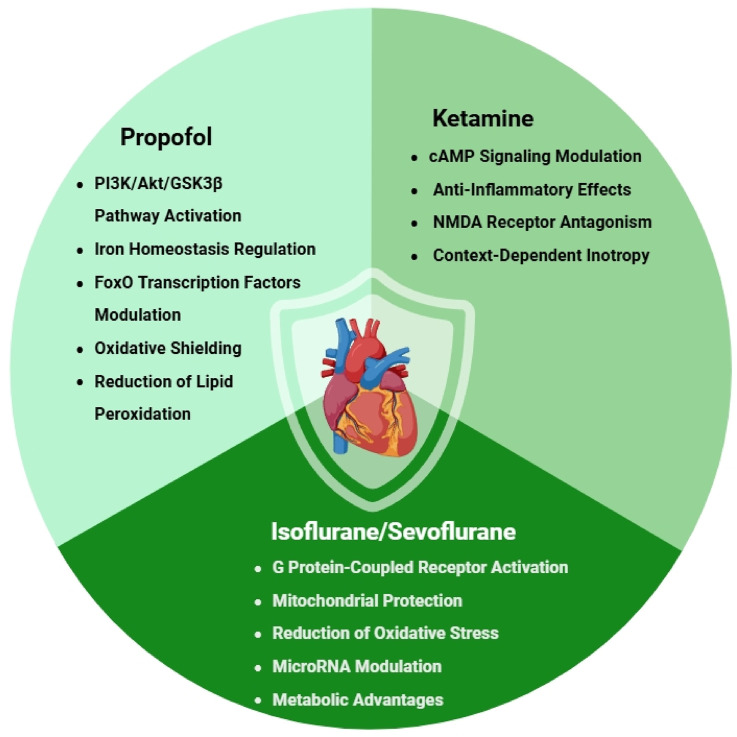
Molecular mechanisms of anesthetic-mediated cardioprotection.

Propofol (2,6-diisopropylphenol) has emerged as one of the most clinically important intravenous anesthetics, with accumulating evidence demonstrating its remarkable cardioprotective properties (Figure 1) (H et al., 2021). Beyond its well-established anesthetic effects, extensive preclinical and clinical investigations have revealed that propofol exerts its protective influence through multiple, intricately interconnected molecular pathways, making it a particularly fascinating subject for ongoing cardiovascular research (Zhao et al., 2015).

The cardioprotective mechanisms of propofol are primarily mediated through activation of the PI3K/Akt/GSK3β signaling pathway, a crucial survival pathway in cardiomyocytes. Detailed mechanistic studies have shown that propofol significantly upregulates Caveolin-3 (Cav-3), an essential membrane scaffolding protein that plays pivotal roles in cardiomyocyte function, signal transduction, and survival. These investigations demonstrated that propofol exerts a protective effect by specifically inhibiting proteasomal degradation of Cav-3 during the critical phases of ischemia-reperfusion injury. This preservation of Cav-3 leads to markedly enhanced activation of the PI3K/Akt/GSK3β pathway (Zhu et al., 2017). The resulting signaling cascade initiates a powerful anti-apoptotic program that promotes cell survival under conditions of ischemic stress, while simultaneously inhibiting key mediators of cell death pathways.

Recent advances in our understanding of propofol’s cardioprotective effects have revealed another significant mechanism involving its regulation of iron homeostasis in cardiomyocytes (Zhang et al., 2019). Given that iron dysregulation and subsequent iron-catalyzed oxidative damage have been strongly implicated in the pathogenesis of ischemia-reperfusion injury (Sun et al., 2024; Pan et al., 2022), these findings take on particular clinical relevance. Cutting-edge research has demonstrated that propofol effectively inhibits pathological iron deposition in both H9c2 cardiomyoblast cells and *in vivo* mouse myocardium through sophisticated modulation of the AKT/p53 signaling pathway (Li et al., 2022). This iron-regulating effect appears to be especially important for reducing the burst of reactive oxygen species (ROS) generation that typically occurs during the reperfusion phase, thereby substantially limiting oxidative damage to critical cellular components including lipids, proteins, and DNA.

Further expanding our understanding of propofol’s multifaceted protective effects, studies have identified its significant influence on FoxO transcription factors, which serve as master regulators of cellular responses to oxidative stress. Comprehensive research has shown that propofol post-conditioning dramatically improves outcomes following hypoxia/reoxygenation-induced injury by reducing apoptosis and modulating autophagy in cardiac cells through upregulation of forkhead transcription factors (H et al., 2021; Zhang et al., 2022). This represents an additional, independent layer of cardioprotection that complements rather than merely duplicates the benefits mediated through the PI3K/Akt pathway.

The intrinsic antioxidant properties of propofol deserve particular emphasis in any discussion of its cardioprotective mechanisms. The drug’s unique chemical structure, featuring a phenolic hydroxyl group, confers exceptional free radical scavenging capabilities that make it particularly effective at mitigating oxidative stress during the critical reperfusion period (Marik, 2005). This direct antioxidant activity synergizes with its effects on cellular signaling pathways to create a comprehensive, multifaceted protective profile that addresses multiple aspects of ischemia-reperfusion injury (Hausburg et al., 2020). Rigorous experimental studies have consistently shown that propofol treatment reduces markers of lipid peroxidation, helps preserve mitochondrial membrane potential integrity, and maintains optimal cellular glutathione levels during ischemia-reperfusion scenarios (Ranjbar et al., 2014; Yoo et al., 1999; Xia et al., 2004; Shao et al., 2008).

Perhaps most intriguingly, clinical and experimental observations have revealed that propofol’s cardioprotective effects follow a clear dose-response relationship (Shao et al., 2008; Vanlersber et al., 2008). While moderate, clinically relevant doses provide significant protection against ischemia-reperfusion injury, very high concentrations may paradoxically produce negative inotropic effects and impair cardiac function, particularly in immature or developing hearts. This biphasic dose-response curve, first systematically characterized in preclinical models, carries important implications for optimizing dosing strategies across different patient populations and clinical scenarios (Shirakawa et al., 2014). The recognition of this dose-dependent effect has prompted more nuanced approaches to propofol administration in cardiac patients and those at risk of perioperative ischemia.

### 2.2 Ketamine

Ketamine has emerged as a uniquely versatile intravenous anesthetic with multifaceted cardioprotective properties that distinguish it from other agents in its class (Figure 1) (Trimmel et al., 2018; Molojavyi et al., 2001; Hirota and Lambert, 2011; Zanos et al., 2018; Hudetz and Pagel, 2010). While it shares certain protective pathways with propofol, particularly in modulating cellular survival signals (Cope et al., 1997), ketamine’s distinct pharmacological profile - combining NMDA receptor antagonism with sympathomimetic and anti-inflammatory effects - confers specific clinical advantages that may be particularly valuable in high-risk cardiac scenarios (Ko et al., 1997b; Han et al., 2002). Beyond its direct cardioprotective roles, these properties also align with evolving perioperative strategies aimed at modulating surgical stress responses and reducing systemic inflammation through multimodal analgesic approaches (Zanos et al., 2018; Zhang et al., 2024; Jian et al., 2018).

The drug’s cardioprotective mechanisms operate through an integrated network of pathways that address multiple aspects of ischemic and inflammatory myocardial injury. A particularly noteworthy mechanism involves ketamine’s modulation of cAMP signaling, which serves as a crucial second messenger system in cardiomyocytes (Peña and Wolska, 2005). Detailed cellular studies have demonstrated that ketamine not only enhances basal cAMP levels in cardiac cells but also effectively counteracts the pathological suppression of cAMP induced by pro-inflammatory cytokines like TNF-α and IL-1β (Hill et al., 1998). This dual action helps maintain critical intracellular signaling during the inflammatory storms that frequently accompany cardiac surgery and ischemia-reperfusion injury, potentially preserving myocardial contractility and metabolic function when they are most compromised.

Ketamine’s robust anti-inflammatory properties constitute another major component of its cardioprotective arsenal (Natoli, 2021; Ibrahim et al., 2017). The drug exerts a multimodal immunomodulatory effect, simultaneously suppressing the production of damaging pro-inflammatory cytokines (including TNF-α, IL-6, and IL-1β) while enhancing the release of protective anti-inflammatory mediators like IL-10. These effects are achieved through several complementary mechanisms: direct inhibition of the NF-κB signaling pathway (a master regulator of inflammatory gene expression), modulation of the NLRP3 inflammasome complex, and potential effects on toll-like receptor signaling (Bi et al., 2024; Santos et al., 2025). Such comprehensive anti-inflammatory activity may be especially beneficial in clinical conditions where systemic inflammation directly contributes to myocardial dysfunction, such as in sepsis-induced cardiomyopathy, post-cardiac arrest syndrome, or the systemic inflammatory response following cardiopulmonary bypass (Zhang et al., 2021).

The NMDA receptor antagonism that forms the basis of ketamine’s anesthetic and analgesic properties also contributes meaningfully to its cardioprotective profile (Tyagi et al., 2009). During ischemic episodes, excessive glutamate release leads to sustained activation of myocardial NMDA receptors (particularly those containing GluN2B subunits), resulting in pathological calcium influx and subsequent activation of cell death pathways (Abbaszadeh et al., 2018). Ketamine’s potent blockade of these receptors helps break this vicious cycle, reducing calcium-mediated injury during both the ischemic and reperfusion phases (Lisek et al., 2020; Iacobucci and Popescu, 2024). Interestingly, this mechanism may complement the drug’s other protective effects, as NMDA receptor overactivation has been linked to both inflammatory signaling and oxidative stress in cardiomyocytes.

Ketamine’s effects on myocardial contractility present a fascinating paradox that underscores the context-dependent nature of its actions (Kunst et al., 1999). While the drug can produce mild negative inotropic effects in healthy myocardium (likely through L-type calcium channel modulation), numerous clinical observations suggest it may actually better preserve ventricular function in failing or stressed hearts compared to alternative anesthetics (Hanouz et al., 2004; Sprung et al., 1998). This apparent paradox may reflect ketamine’s unique ability to maintain sympathetic tone while simultaneously providing cellular protection against ischemia and inflammation - a combination particularly suited to compromised myocardium.

The convergence of these diverse mechanisms - spanning metabolic regulation, inflammatory control, receptor modulation, and functional preservation - establishes ketamine as an exceptionally versatile cardioprotective agent. Its multifaceted action profile makes it particularly valuable in complex clinical scenarios where myocardial ischemia coexists with systemic inflammation, autonomic instability, or pre-existing cardiac dysfunction. Looking forward, important research priorities include the development of optimized dosing protocols for specific high-risk populations (such as patients with septic cardiomyopathy or advanced heart failure), investigation of potential synergistic effects when combined with other cardioprotective strategies (including remote ischemic preconditioning or targeted temperature management), and exploration of its role in emerging applications like donor heart preservation for transplantation.

### 2.3 Volatile anesthetics

Volatile anesthetics, including isoflurane, sevoflurane, and desflurane, have been extensively documented to exert significant cardioprotective effects across both preclinical and clinical investigations (Van Allen et al., 2012; Tanaka et al., 2004; Pratt et al., 2006; Suda and Uka, 2022). These pharmacological agents mimic the protective mechanisms of ischemic preconditioning while offering the distinct advantage of being pharmacologically inducible without necessitating actual ischemic events (Hara, 2006; Riess et al., 2004). The cardioprotective properties of volatile anesthetics are mediated through multifaceted interactions involving diverse cellular targets (Figure 1) (Agarwal et al., 2014a; Kikuchi et al., 2015; Zhang T. et al., 2025), with their effects being particularly pronounced when administered during the preconditioning phase.

Isoflurane demonstrates comprehensive cardioprotection through several synergistic mechanisms: substantial reduction of oxidative stress, preservation of mitochondrial structural and functional integrity, and optimization of intracellular calcium homeostasis (An et al., 2007; Agarwal et al., 2014b; Liu and Liu, 2018; Lang et al., 2013). Experimental evidence has demonstrated that isoflurane pretreatment significantly attenuates cardiac oxidative damage following ischemia-reperfusion injury, as quantified by reductions in established biomarkers of lipid peroxidation and protein oxidation (Li et al., 2012). This antioxidant effect is mediated through both direct free radical scavenging and upregulation of endogenous antioxidant defense systems. The understanding of isoflurane’s cardioprotective mechanisms has been substantially advanced by recent discoveries in molecular cardiology. Research has revealed that isoflurane exerts its protective effects not only through immediate pharmacological actions but also by inducing lasting epigenetic modifications. Studies examining cardiac cells exposed to hypoxia/reoxygenation injury demonstrate that isoflurane pretreatment significantly alters microRNA-363-3p expression patterns (Ge et al., 2022). This microRNA modulation initiates a comprehensive cellular defense program that simultaneously regulates apoptotic pathways through Bcl-2 family proteins, enhances antioxidant defenses via Nrf2 pathway activation, and strengthens pro-survival signaling through Akt and ERK pathway modulation. These findings fundamentally expand our comprehension of volatile anesthetic-mediated protection by demonstrating its capacity to establish persistent epigenetic changes that confer myocardial resilience. The sustained alteration of microRNA expression profiles provides a molecular basis for the long-lasting cardioprotective effects observed following isoflurane exposure, extending well beyond the acute perioperative period.

Sevoflurane has emerged as a clinically promising cardioprotective agent, exerting its effects through multiple synergistic mechanisms. The anesthetic enhances myocardial ischemic tolerance primarily by activating protein kinase C (PKC) and mitochondrial ATP-sensitive potassium (KATP) channels, thereby preserving mitochondrial function during ischemia-reperfusion injury (Hara et al., 2001; Jiang et al., 2016; Bouwman et al., 2007; de Ruijter et al., 2003; Bouwman et al., 2006). During ischemic conditioning procedures, sevoflurane exposure induces significant molecular changes in cardiomyocytes, including upregulation of cardioprotective microRNAs and cytokines, while simultaneously suppressing mediators of cellular damage (Guerrero-Orriach et al., 2024). Additionally, sevoflurane exerts cardioprotective effects against hypoxia/reoxygenation-induced myocardial injury by downregulating lncRNA LINC00265, which functions as a molecular sponge to inhibit miR-370-3p, thereby reducing apoptosis, and inflammatory cytokine release (IL-6, TNF-α) (Shao et al., 2025). Notably, sevoflurane preconditioning activates the PI3K/AKT/GSK3β pathway to upregulate Syntaxin1a, significantly reducing myocardial apoptosis in murine ischemia-reperfusion models (Liu et al., 2025). These multi-targeted actions - encompassing ion channel modulation, kinase signaling activation, and epigenetic regulation - collectively contribute to sevoflurane’s superior clinical performance in cardiac protection, particularly in surgical preconditioning protocols and potential applications for acute coronary syndromes.

These molecular effects translate to measurable clinical benefits, as demonstrated in randomized trials showing sevoflurane’s superiority over propofol in patients undergoing cardiopulmonary bypass. Specifically, sevoflurane-treated patients exhibit reduced postoperative troponin release and improved ventricular functional recovery (Marcos-Vidal et al., 2014; Likhvantsev et al., 2016). Its rapid onset/offset pharmacokinetics make it especially suitable for preconditioning protocols (Beukers et al., 2025). Desflurane, while less studied, shows comparable cardioprotective potential (Qin and Zhou, 2023; Ozarslan et al., 2012). Its low blood solubility allows precise titration, and evidence suggests it may be particularly effective when administered during early reperfusion. However, its strong sympathetic activation effects require careful hemodynamic management (Landoni et al., 2007).

Volatile anesthetics also appear to influence myocardial metabolism in ways that may enhance ischemic tolerance (van den Brom et al., 2013; Stowe and Kevin, 2004). Several studies have reported that these agents promote a shift toward more efficient energy utilization during ischemia, potentially by modulating substrate selection and improving mitochondrial coupling. These metabolic effects may complement the direct protective actions on signaling pathways and ion channels (Stowe and Kevin, 2004; Yamanaka and Hayashi, 2009; Zhang et al., 2023). Beyond their metabolic effects, volatile anesthetics modulate ion transporter activity, notably that of the K^+^-Cl^-^ cotransporter 2 (KCC2). While KCC2 has traditionally been studied for its role in maintaining neuronal chloride homeostasis and facilitating emergence from anesthesia (Hu et al., 2023; Song and Hu, 2024), emerging evidence also supports its functional significance in the heart (Modi et al., 2023). It is thus hypothesized that during ischemia-reperfusion injury, volatile anesthetics may influence cardiac KCC2 activity, thereby potentially contributing to the regulation of cell volume and ionic balance. Importantly, the cardioprotective benefits of volatile anesthetics may be significantly enhanced through synergistic combination with lung-protective ventilation strategies. By employing lower tidal volumes, optimal PEEP, and careful avoidance of hyperoxia, anesthesiologists can reduce ventilator-induced lung injury and the subsequent inflammatory cross-talk between the lung and heart, thereby creating a more favorable environment for the myocardial protective effects of volatile agents to manifest (Zou et al., 2024; Ferrando et al., 2015). Clinical implementation continues to evolve, with current evidence supporting volatile use throughout cardiac procedures rather than limited to preconditioning phases (Bonanni et al., 2020). Ongoing research explores optimal combinations with other protective strategies and applications in non-cardiac surgeries for high-risk patients (Zhang et al., 2023; Park et al., 2020).

## 3 Comparative cardioprotective efficacy of anesthetic agents

### 3.1 Intravenous vs. volatile anesthetics

The comparative cardioprotective efficacy between intravenous and volatile anesthetic agents has been extensively studied in both laboratory and clinical settings, with a primary focus on patients undergoing cardiac surgery (Bonanni et al., 2020; Bein et al., 2005). A key clinical trial evaluating propofol-based total intravenous anesthesia versus sevoflurane anesthesia in coronary artery bypass graft (CABG) procedures demonstrated better cardioprotection with the volatile anesthetic, as indicated by improved cardiac functional parameters and lower troponin release (De Hert et al., 2002). Subsequent comprehensive reviews and pooled analyses have largely supported these initial findings, showing a consistent, albeit modest, cardioprotective benefit favoring volatile agents in cardiac surgical contexts. However, a more detailed analysis reveals several important considerations. Propofol appears to be particularly effective when used as a postconditioning intervention rather than as the primary maintenance anesthetic (He et al., 2008; Liu et al., 2021). Growing evidence indicates that combining propofol with volatile anesthetics may produce enhanced protective effects, although the ideal dosing protocols for such combined approaches need further clarification (Schumacher et al., 2009; Diz et al., 2010; Wolf et al., 2021). These findings highlight the critical need for selecting anesthetic techniques based on specific clinical circumstances to optimize myocardial protection during cardiac procedures.

The differential effects likely stem from distinct molecular mechanisms: volatile anesthetics primarily act through preconditioning pathways involving mitochondrial KATP channels and protein kinase C activation, while propofol’s benefits are more related to its antioxidant properties and direct effects on cellular survival pathways. This mechanistic diversity suggests potential advantages for either approach depending on the specific clinical scenario and patient characteristics.

### 3.2 Age and comorbidity considerations

The effectiveness of anesthetic-mediated cardioprotection demonstrates significant variability across different patient populations, with age and comorbid conditions emerging as key determinants of therapeutic response (van den Brom et al., 2013; Ruiz-Meana et al., 2020; Domene et al., 2025). Developmental stage represents a particularly important consideration, as evidenced by research demonstrating heightened vulnerability of immature myocardium to potential adverse effects at elevated propofol concentrations (Shirakawa et al., 2014). This age-dependent sensitivity may reflect differences in drug metabolism, receptor expression patterns, or cellular stress responses during cardiac development.

Advanced age similarly influences anesthetic cardioprotection, with experimental studies showing attenuated protective effects in senescent animal models for both volatile anesthetics and propofol (Ruiz-Meana et al., 2020; Suleiman et al., 2015; Sniecinski and Liu, 2004). The underlying mechanisms likely involve age-associated alterations in cellular signaling cascades, mitochondrial bioenergetics, and redox homeostasis that collectively impair preconditioning responses. These findings have important implications for geriatric patients undergoing cardiac procedures. Diabetes mellitus constitutes another critical modifier of anesthetic cardioprotective efficacy (Liu and Xia, 2012; Drenger et al., 2011; Canfield et al., 2016). The pathological hyperglycemic milieu appears to disrupt multiple protective signaling pathways, including those mediated by adenosine receptors and protein kinase C isoforms. Clinical observations parallel preclinical findings, demonstrating reduced cardioprotection in diabetic patients that may necessitate modified anesthetic strategies (Amour et al., 2010; Canfield et al., 2012). Potential approaches include tighter perioperative glycemic control or the use of adjunctive agents to restore protective signaling.

These collective findings emphasize the necessity of developing personalized anesthetic regimens that carefully consider multiple patient-specific factors including developmental stage and chronological age, the presence and severity of metabolic comorbidities (particularly renal and hepatic function), baseline myocardial function, and the anticipated surgical stress and ischemic burden (Zhu et al., 2024; Abraham et al., 2023; Zhu et al., 2023). Current research in this area would benefit from incorporating large-scale human genetic and epidemiological insights to better understand patient heterogeneity and refine personalized approaches (Chen et al., 2025; Zhu SQ. et al., 2025; Yurkovich et al., 2024). Such comprehensive patient profiling enables clinicians to optimize cardioprotection while minimizing potential adverse effects, particularly in vulnerable populations where standard anesthetic approaches may prove less effective. The integration of these considerations into clinical decision-making represents an important step toward precision medicine in perioperative cardioprotection, requiring careful evaluation of how age-related physiological changes, comorbid conditions, and procedural factors interact to influence anesthetic efficacy. Future research should focus on developing validated clinical algorithms that systematically incorporate these multidimensional patient characteristics to guide anesthetic selection and dosing in cardiac surgery populations.

## 4 Clinical applications and challenges

### 4.1 Cardiac surgery applications

The most compelling clinical application of anesthetic cardioprotection lies in cardiac surgery, where ischemia-reperfusion injury remains an unavoidable consequence of cardiopulmonary bypass and aortic cross-clamping (Torregroza et al., 2020; Landoni et al., 2019). A substantial body of evidence now demonstrates that anesthetic selection can significantly impact key postoperative outcomes, including ventricular function recovery, incidence of arrhythmias, and magnitude of cardiac enzyme release (Uhlig et al., 2016). Volatile anesthetics, particularly sevoflurane and desflurane, have emerged as preferred agents in many cardiac centers, with numerous studies demonstrating their superiority over propofol-based total intravenous anesthesia in preserving myocardial function following coronary artery bypass grafting (CABG) (De Hert et al., 2002; Zangrillo et al., 2015; Zhang Y. et al., 2025). Meta-analyses of randomized controlled trials consistently show approximately 20%–30% reductions in troponin release with volatile-based regimens, along with improved early postoperative ejection fraction and reduced inotropic requirements (Yu and Beattie, 2006; Li and Yuan, 2015). The protective effects appear most pronounced in isolated CABG procedures, where ischemic times are typically shorter, though benefits have also been documented in more complex valve surgeries requiring longer cardioplegic arrest (Zangrillo et al., 2022; Piriou et al., 2000). Mechanistically, these clinical observations align with laboratory findings demonstrating volatile anesthetics’ ability to preserve mitochondrial function, reduce oxidative stress, and attenuate calcium overload during reperfusion (De Hert et al., 2008). However, the implementation of volatile-based cardiac anesthesia requires careful consideration of several practical factors, including the need for specialized vaporizers in the bypass circuit and potential interactions with cardioplegia solutions (Landoni et al., 2013). Furthermore, the optimal dosing strategy - whether to administer volatiles throughout surgery or concentrate exposure during specific preconditioning or postconditioning phases - remains an active area of investigation (Meybohm et al., 2015). Interestingly, beyond direct pharmacological conditioning, emerging evidence suggests that regional analgesic techniques such as novel fascial plane blocks (e.g., erector spinae plane or parasternal blocks) may also contribute to systemic anti-inflammatory and cardioprotective effects by modulating neuroimmune pathways and reducing surgical stress responses (Bagnol et al., 2024; Sandeep et al., 2022). This multimodal approach—combining volatile anesthetics with regional techniques—may offer complementary benefits for cardioprotection. Recent studies suggest that combining volatile anesthetics with remote ischemic preconditioning may provide additive protective benefits (Chen et al., 2022), while others have explored the potential of pharmacologic postconditioning with volatile agents during the critical reperfusion period (Lurati Buse et al., 2012). The ongoing debate about the clinical significance of these protective effects continues to drive research into optimal anesthetic protocols for various cardiac surgical populations (Yu, 2011).

### 4.2 Expanding applications in non-cardiac surgery

While the cardioprotective effects of anesthetics are most extensively studied in cardiac surgery, emerging evidence suggests potential applications in high-risk non-cardiac procedures (Van Allen et al., 2012; Landoni et al., 2009a). Vascular surgery patients, who frequently have significant underlying coronary disease, may represent a particularly promising population for anesthetic-mediated protection (Bas et al., 2012). Preliminary studies indicate that volatile anesthetic use during major vascular procedures may reduce postoperative cardiac complications by 15%–25%, though these findings require confirmation in larger, multicenter trials (Landoni et al., 2009a). The physiological rationale for this protection stems from the frequent hemodynamic fluctuations and potential ischemic episodes during vascular surgery, creating conditions where anesthetic preconditioning could theoretically attenuate myocardial stunning (Harris, 2022; Chen et al., 2018). Other surgical populations that might benefit include patients undergoing major orthopedic procedures (who often have cardiovascular comorbidities) (Kvarda et al., 2022) and those receiving solid organ transplants (where ischemia-reperfusion injury affects both the graft and potentially the heart) (Eltzschig and Eckle, 2011; Jahn et al., 2022). However, several unique challenges arise when considering anesthetic cardioprotection outside cardiac surgery (Landoni et al., 2009b; Hovaguimian et al., 2014). First, the duration and magnitude of ischemic insults are typically less predictable than in controlled cardiac procedures, making optimal timing of protective strategies more difficult (Kalogeris et al., 2012; Zaugg et al., 2014). Second, the balance between potential cardiac benefits and other considerations (such as effects on cerebral or renal perfusion) becomes more complex in heterogeneous non-cardiac surgeries (Stoppe et al., 2017; Petersen et al., 2018). Third, practical constraints like operating room workflow and equipment availability may limit volatile anesthetic use in some non-cardiac settings (Habte et al., 2024). Despite these challenges, the high incidence of perioperative cardiac events in vulnerable populations continues to drive interest in expanding anesthetic cardioprotection strategies beyond traditional cardiac surgery applications (Landoni et al., 2009b; Wong and Irwin, 2016). Future research should focus on identifying which non-cardiac surgical patients stand to benefit most (Hong et al., 2019), developing protocols that integrate seamlessly with diverse surgical workflows (Schild et al., 2019), and determining whether brief exposure to protective anesthetics (rather than maintenance throughout surgery) might suffice for risk reduction (Yoo et al., 2024; Orriach et al., 2020).

### 4.3 Translational challenges

**FIGURE 2:**
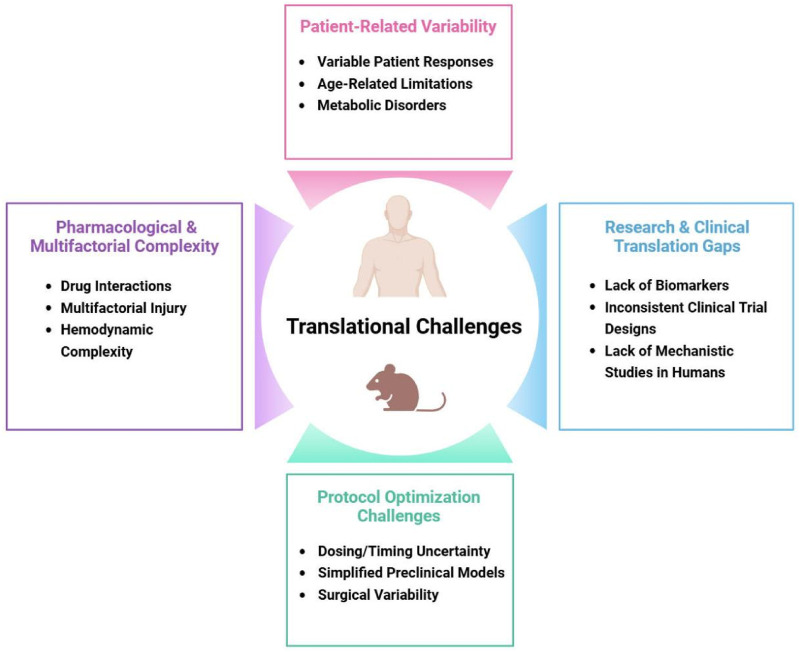
Challenges in translating anesthetic-induced cardioprotection from bench to bedside.

Despite compelling preclinical evidence demonstrating the cardioprotective potential of various anesthetics (Li et al., 2024), translating these findings into consistent clinical benefits has proven challenging (Figure 2) (Lin et al., 2021; Pagel, 2009). One major obstacle is the significant variability in patient responses to protective strategies, influenced by factors such as age, genetic background, and comorbidities like diabetes or heart failure (Kikuchi et al., 2015). For instance, studies suggest that the efficacy of anesthetic preconditioning may be attenuated in elderly or diabetic patients due to age-related mitochondrial dysfunction or metabolic disturbances that impair protective signaling pathways (Mio et al., 2008). Another critical challenge lies in determining the optimal dosing and timing protocols for anesthetic-induced cardioprotection (Frässdorf et al., 2009). While animal studies often use standardized ischemia-reperfusion models (Alemany et al., 2023), clinical scenarios present complex variables including differing surgical durations (Glance et al., 2018), varying ischemic insults (Sandroni et al., 2021), and heterogeneous patient physiologies (See, 2023) that complicate protocol standardization (Kahol et al., 2011; Zho et al., 2016). Additionally, the interactions between anesthetic agents and other perioperative medications—such as beta-blockers, statins, or vasopressors—may either potentiate or interfere with cardioprotective mechanisms (Riess, 2009), adding another layer of complexity to clinical application. Perhaps most fundamentally, the multifactorial nature of perioperative myocardial injury means that anesthetic strategies alone cannot address all potential contributors to cardiac damage (Priebe, 2005), including surgical trauma, systemic inflammation, and hemodynamic instability (Chiari and Fellahi, 2024). These translational gaps highlight the need for more sophisticated clinical research approaches that account for real-world variability while maintaining scientific rigor.

## 5 Future directions

To overcome current limitations and fully realize the clinical potential of anesthetic-mediated cardioprotection, several key research directions should be prioritized. First, there is an urgent need to develop validated biomarkers—using genomic, proteomic, or metabolomic profiling—that can identify patients most likely to benefit from specific anesthetic strategies. Integrating these biomarkers with emerging perioperative precision monitoring platforms, such as continuous hemodynamic and metabolic tracking, would enable dynamic, physiology-guided personalization of anesthetic regimens tailored to individual patient characteristics and real-time surgical demands. Second, investigating rational combinations of anesthetics—such as pairing volatile agents with propofol or adjuncts like dexmedetomidine—may yield synergistic effects that maximize protection while minimizing adverse outcomes; this approach should be systematically evaluated in both preclinical models and clinical trials. Third, exploring non-anesthetic drugs that target the same protective pathways (e.g., PI3K/Akt activators or mPTP inhibitors) could lead to novel cardioprotective therapies applicable to non-surgical settings like acute coronary syndromes. Finally, large-scale, multicenter clinical trials employing standardized outcome measures are essential to establish evidence-based protocols for different patient populations and surgical contexts. These trials should incorporate advanced monitoring techniques to assess cardioprotection in real-time and employ long-term follow-up to evaluate lasting clinical benefits. Importantly, as recent advances highlight the need to integrate organ protection with patient-centered recovery outcomes, future studies should simultaneously evaluate both cardioprotective efficacy and functional recovery metrics—as demonstrated in non-cardiac settings where anesthetic selection directly influences quality of recovery—to comprehensively optimize perioperative care (Zhu S. et al., 2025). By addressing these priorities, future research can bridge the gap between promising laboratory findings and meaningful improvements in patient care.

## 6 Conclusion

The cardioprotective effects of anesthetics represent an exciting convergence of anesthesiology and cardiovascular science. While challenges remain in translating these effects into consistent clinical benefits, the accumulated evidence strongly supports the concept that anesthetic choice can meaningfully influence cardiac outcomes. As our understanding of the underlying mechanisms continues to grow, so too will our ability to harness these effects for patient benefit.
